# Evaluation of expert rules for carbapenemase class identification in *Enterobacterales* isolates using the VITEK2 susceptibility testing platform

**DOI:** 10.1128/jcm.00769-25

**Published:** 2025-09-19

**Authors:** Michaela J. Eickhoff, Abigail P. Brown, Carol E. Muenks, Megan L. Porter, Murad Ali, Iftikhar Uddin, Tahir Hussain, Melanie L. Yarbrough, Rebekah E. Dumm

**Affiliations:** 1 Department of Pathology and Immunology, Washington University School of Medicine 12275 , St. Louis, Missouri, USA; 2 Mardan Medical Complex, Mardan, Pakistan; 3 Department of Microbiology, Abdul Wali Khan University Mardan 230180, Mardan, Pakistan; Cleveland Clinic, Cleveland, Ohio, USA

**Keywords:** MDRO, bacteriology, automated AST, antimicrobial susceptibility testing

## Abstract

Prompt identification of enzyme class in carbapenemase-producing carbapenem-resistant *Enterobacterales* (CP-CRE) has critical implications for informing appropriate patient treatment, infection prevention, and public health. This study evaluates the performance of prototype bioMérieux Advanced Reporting Tool (bioART) expert rules to rapidly identify *Klebsiella pneumoniae* carbapenemase (KPC), metallo-β-lactamase (MBL), and OXA-48-like carbapenemase enzymes in CP-CRE. The bioART rules are designed for use with routine cards on the VITEK2 automated antimicrobial susceptibility testing (AST) platform. Results provided by the bioART rules should be interpreted in combination with the instrument Advanced Expert System (AES) to comprehensively evaluate phenotypic resistance phenotypes. Two hundred clinical isolates with varied β-lactam resistance profiles were enrolled, with 196 ultimately analyzed, including 115 CP-CREs. The AES alone detected CP-CRE isolates with a sensitivity of 83% and a specificity of 85%. The combined AES and bioART rules detected CP-CRE with a sensitivity of 96% and a specificity of 83%. Prediction of carbapenemase classes by the bioART rules was analyzed for a subset of 158 isolates, including only the species for which the rules have claimed indications. MBL, KPC, and OXA-48-like enzymes were detected with sensitivities of 97%, 76%, and 47%, respectively. Notably, sensitivity was 90% for single OXA-48-like producers, whereas OXA-48-like enzymes in dual New Delhi metallo-β-lactamase (NDM)/OXA-48-like-producing isolates were undetectable using phenotypic susceptibility patterns, and isolates were reported only as producing NDM enzymes. Overall, laboratories incorporating these tools into carbapenemase screening workflows may consider their utility in prompting confirmatory carbapenemase testing, guiding modifications to AST reporting, and/or prompting additional susceptibility testing.

**IMPORTANCE:**

Carbapenem-resistant bacteria are a major public health concern due to their ability to spread in healthcare settings and cause infections that are difficult to treat with first-line antibiotics. Identification of the enzyme classes responsible for carbapenem resistance plays a crucial role in ensuring that patients receive effective treatments and controlling the spread of these bacteria. In this study, we evaluated the performance of a new approach to identify carbapenemase enzymes without additional hands-on testing. The method is designed for use with the VITEK2 automated susceptibility testing platform to recognize patterns of resistance to antibiotics and make predictions about the possible resistance mechanisms.

## INTRODUCTION

Carbapenem-resistant *Enterobacterales* (CRE) pose an urgent public health threat due to their ability to spread within healthcare settings and cause infections that are difficult to treat, leading to significant morbidity and mortality (1, 2). Broadly, carbapenem resistance can result from enzymatic hydrolysis of carbapenems by carbapenemases, or non-enzymatic mechanisms, such as overexpression of efflux pumps or mutations in outer membrane porin genes (3). As such, CRE are classified as either carbapenemase-producing carbapenem-resistant *Enterobacterales* (CP-CRE) or as non-carbapenemase-producing CRE (non-CP-CRE). In the United States, the most common carbapenemases identified in CP-CRE include Ambler Class A *Klebsiella pneumoniae* carbapenemase (KPC) enzymes, Ambler Class B New Delhi metallo-β-lactamase (NDM) enzymes, and Ambler Class D oxacillinase (OXA-48-like) enzymes, with prevalence and endemicity varying by region (1). Other, less commonly identified, carbapenemase enzymes include *Serratia marcescens* enzyme (SME), imipenem-hydrolyzing β-lactamase (IMI), and the metallo-β-lactamases (MBLs) Verona integron-encoded metallo-β-lactamase (VIM) and imipenemase (IMP) (4).

Carbapenemase enzymes are often encoded on mobile genetic elements, which can facilitate transmission between bacteria and contribute to nosocomial spread (5). Additionally, the recommended treatment for CP-CRE infections varies by carbapenemase class (6, 7). For these reasons, rapid and accurate identification of the enzyme class in carbapenemase-producing isolates has critical implications for informing appropriate patient treatment, hospital infection control, and public health. Some laboratories may not routinely identify carbapenemase classes, and others employ tiered workflows with confirmatory testing that can introduce delays (4). Clinical laboratories are advised to implement workflows to detect carbapenemases; however, several phenotypic and genotypic testing methods are available (4, 8, 9). Each laboratory must determine its preferred screening criteria and testing workflow, considering factors such as patient population, regional epidemiology, laboratory capabilities, test performance, and testing costs (4, 10).

In this study, the performance of novel prototype bioMérieux Advanced Reporting Tool (bioART) expert rules for identification of carbapenemase classes was analyzed. The bioART rules were designed by bioMérieux for use with routine cards on the VITEK2 automated antimicrobial susceptibility testing (AST) system to identify KPC, MBL, and OXA-48-like carbapenemases based on resistance profiles. These rules are currently available for laboratories that choose to implement them. The results of the prototype bioART rules are intended for combined use with the results from the VITEK2 instrument Advanced Expert System (AES). The AES characterizes resistance mechanisms by comparing AST results to a database of minimum inhibitory concentration (MIC) distributions of microbial species with various resistance mechanisms (11, 12). As currently available, the AES can generally predict carbapenemase production and specifically predict “SME-like” carbapenemases. Performance of the AES alone and the AES with the bioART rules was evaluated by performing VITEK2 AST using N802 and XN15 cards on a challenging set of clinical *Enterobacterales* isolates with varied resistance to β-lactam antibiotics, including 115 CP-CREs. The ability of the AES and bioART rules to detect CP-CRE and accurately predict carbapenemase classes was evaluated for the study isolates. Performance was compared to genotypic detection of carbapenemase genes by Xpert Carba-R or whole genome sequencing (WGS). To assess how the AES and bioART rules might contribute to a routine laboratory workflow for carbapenemase detection and characterization, the tools were evaluated for use in (i) prompting confirmatory carbapenemase testing, (ii) guiding modifications to AST reporting, and (iii) prompting additional susceptibility testing.

## MATERIALS AND METHODS

### Study isolates

Two hundred clinical *Enterobacterales* isolates with varied β-lactam resistance profiles were enrolled in the study. One hundred sixty-four of the isolates were collected from 2015 to 2024 from the clinical microbiology laboratory at Barnes Jewish Hospital, a tertiary care medical center in St. Louis, MO. One isolate was a previously characterized IMP-27-producing *Morganella morganii* (13). The remaining 35 *Enterobacterales* isolates were previously collected from hospitalized patients admitted to the medical ward from 2021 to 2022 at Mardan Medical Complex, a tertiary care hospital in Pakistan. A list of study isolates is provided (Table S1).

### VITEK2 antimicrobial susceptibility testing

Study isolates were subcultured twice from frozen (−80°C) stocks before AST was performed: first, to a MacConkey agar plate with a meropenem (MEM) disk, and second, growth around the meropenem disk was subcultured to a blood agar plate to confirm purity. Identification of each isolate was confirmed using the VITEK MS PRIME MALDI-TOF mass spectrometry system utilizing Knowledge Base v.3.2 (bioMérieux, La Balme-les-Grottes, France) prior to testing, and these identifications were used as the final enrollment identification (Table S1). AST was performed according to manufacturer’s instructions on 24 h growth from the blood agar plates using the N802 and XN15 VITEK2 test cards. Testing with both cards was performed simultaneously by inoculation using the same McFarland dilution on the VITEK2 060 instrument utilizing the VITEK 2 AES analysis v.9.03.3 (bioMérieux). A blood agar plate was set up in tandem to confirm purity. Per the package insert, MIC values were interpreted by the VITEK2 system according to the 2017 Clinical and Laboratory Standards Institute (CLSI) M100-S27 guideline (14). Prototype bioART rules for carbapenemase class detection were installed on the VITEK2 system for detection of KPC, MBL, and OXA-48-like carbapenemases. The prototype bioART rules were designed by bioMérieux and are proprietary. Carbapenemase class predictions are based on resistance profiles to piperacillin-tazobactam, cefotaxime, ceftazidime, ceftriaxone, cefepime, aztreonam, imipenem, ertapenem, meropenem, meropenem-vaborbactam (MEV), ceftazidime-avibactam, and ceftolozane-tazobactam; however, results from bug/drug combinations that are not Food and Drug Administration (FDA)-cleared on the VITEK2 cards are not utilized. The bioART rules for carbapenemase detection are intended for use with *Enterobacterales* species but do not have claimed indications for all members of this group, including *Proteus*, *Providencia*, *Morganella*, and *Citrobacter* spp. other than *Citrobacter freundii* complex. The prototype bioART rules are currently available to clinical laboratories for immediate implementation; the bioMérieux Field Application Specialist team may be contacted for details.

### Enrollment criteria for isolate mechanism of resistance

The mechanism of beta-lactam resistance for each isolate was categorized based on a composite characterization by phenotypic VITEK2 AST, modified carbapenem inactivation method (mCIM) (15) and Xpert Carba-R genotypic detection of KPC, NDM, VIM, OXA-48, and IMP genes (Cepheid, Sunnyvale, CA). mCIM was performed for all isolates that tested intermediate or resistant to any carbapenem by VITEK2 AST, and the Xpert Carba-R was performed for all mCIM(+) isolates. Isolates that tested susceptible to all third-generation cephalosporins and carbapenems by the VITEK2 N802 and XN15 cards (ceftazidime, ceftriaxone, cefotaxime, cefpodoxime, meropenem, ertapenem, and imipenem) were enrolled as wild type (WT). 4 *Proteus* isolates that tested intermediate to imipenem but were mCIM(−) were also enrolled as WT. Isolates that tested by VITEK2 AST as resistant to one or more third-generation cephalosporins but susceptible or intermediate to all carbapenems and were mCIM(−) when indicated were enrolled as extended-spectrum β-lactamase (ESBL)/AmpC β-lactamase (AmpC). CRE isolates were defined as isolates that tested resistant to one or more carbapenems by VITEK2 AST and/or as mCIM(+). CP-CREs were differentiated from non-CP-CRE by mCIM, and carbapenemase detection by the Carba-R or WGS defined the carbapenemase class enrollment. Table S1 lists the enrollment criteria for all study isolates.

### Discrepancy analysis

Discrepant analyses for isolate enrollment criteria were performed for isolates in which carbapenemase detection by Carba-R, mCIM, and/or VITEK2 AST did not match expected results. Discrepant analyses are described in Table S1. Briefly, four mCIM(+) isolates did not have a carbapenemase gene detected by the Xpert Carba-R. WGS performed on the isolates identified three to encode an SME and one to encode IMI-1. The enrollment criteria of these four isolates were updated based on the WGS results. Two additional isolates (one *Enterobacter cloacae* complex and one *Serratia marcescens*) that were mCIM(+) did not have a carbapenemase gene detected by WGS. The mCIM(+) results were considered false positives, which have been shown to rarely occur for AmpC-producing isolates (15). A total of four isolates were excluded from the study analyses: two due to mixed culture results and two due to poor growth on the VITEK2 AST cards, resulting in incomplete data. A total of 196 isolates were included in the study analyses (Table S1).

### Genomic characterization of carbapenemases by WGS

WGS was performed for genotypic detection of carbapenemase genes for isolates with unexpected Carba-R, mCIM, and/or VITEK2 AST, described above. The isolates were grown to logarithmic phase in CAMHB and pelleted. Genomic DNA was isolated using a DNeasy UltraClean 96 Microbial Kit (Qiagen, Germantown, MD) according to manufacturer’s instructions. Libraries were prepared from 100 ng of DNA using an Illumina DNA prep kit, normalized, and pooled. Pooled libraries were sequenced on a NextSeq 1000 (Illumina, San Diego, CA) using an Illumina P2 kit. Paired-end reads were assembled and assessed for quality using the AQUAMIS pipeline v.1.4.1 (16). To identify carbapenem resistance mechanisms, assemblies were analyzed by Resistance Gene Identifier (RGI) v.6.0.3, using the Comprehensive Antibiotic Resistance Database v.3.3.0 as a source of reference genes and annotations (17). To identify carbapenem resistance mechanisms, RGI results were filtered to include only mechanisms containing “carbapenem” in the “Drug Class” annotation column. WGS results are listed in Table S1.

### Interpretations of combined AES and prototype bioART rule results

Twenty-four distinct AES reports were categorized into four groups: WT, ESBL/AmpC-Producing ± impermeability, carbapenemase-producing ± impermeability, and inconsistent (Table S2). An “inconsistent” AES report indicates that the susceptibility pattern of the isolate did not match a known phenotype within the AES database. The bioART rules for carbapenemase class prediction do not have claimed indications for all *Enterobacterales* species. Thirty-eight isolates (16 *Proteus* spp., 13 *Citrobacter* spp. other than *Citrobacter freundii* complex, 6 *Morganella* spp., 2 *Providencia* spp., and 1 *Kluyvera* spp.) are not claimed by the bioART rules and were not included in the bioART performance analyses. For the 158 claimed isolates, the AES and bioART rule results were interpreted in combination for carbapenemase class detection. For example, if the AES predicted an isolate as an ESBL/AmpC, a bioART prediction of the possibility of a KPC carbapenemase was disregarded. The isolate was interpreted as a possible ESBL/AmpC producer. Alternatively, if the AES predicted carbapenemase production and the bioART predicted the possibility of a KPC carbapenemase, the isolate was interpreted as a possible KPC producer. In cases where the AES report was inconsistent, the bioART prediction of a possible KPC carbapenemase was trusted, and the isolate was interpreted as a possible KPC producer. A summary of the combined interpretations of AES and bioART reports is provided along with the number of isolates for which each scenario applied (see Table 3).

### Laboratory workflow analysis

The bioART rules for carbapenemase detection are available to clinical laboratories; however, they are not FDA-cleared. Instead, they are intended to support internal laboratory decision-making. To assess the impacts of the AES and bioART rules on laboratory workflow, three potential actions laboratories may take based on the results were assessed: confirmatory carbapenemase testing, modifications to AST reporting, and performance of additional AST (Fig, S1). In the first case, if carbapenemase production was predicted by the AES and bioART rules, regardless of carbapenemase class, confirmatory carbapenemase testing was prompted. Next, the AES and bioART rules were analyzed as tools to prompt modifications to AST reporting. Specifically, in agreement with CLSI guidance, susceptible or susceptible dose-dependent cefepime results were prompted to be suppressed or reported as resistant for all isolates predicted as CP-CRE (18). Also, susceptible or intermediate meropenem-vaborbactam results were prompted to be suppressed or reported as resistant for isolates predicted to produce an OXA-48-like enzyme (18). Lastly, the AES and bioART rules were analyzed as a tool to prompt additional AST. Prediction of a KPC carbapenemase was used to prompt susceptibility testing of imipenem-relebactam, and prediction of a MBL carbapenemase was used to prompt susceptibility testing of cefiderocol and/or aztreonam-avibactam (7). The laboratory workflow analyses were performed on the entire study cohort of 196 isolates, and the laboratory actions were guided by the AES + bioART interpretations that incorporated MEM/MEV MIC ratios (Table S3). For the 38 isolates without claimed indications for the bioART rules, workflow decisions were determined based on the AES reports. 2024 CLSI breakpoints for cefepime were applied to the VITEK2 MIC data for this analysis (18).

## RESULTS

### Laboratory workflow considerations for CP-CRE detection

**Fig 1:**
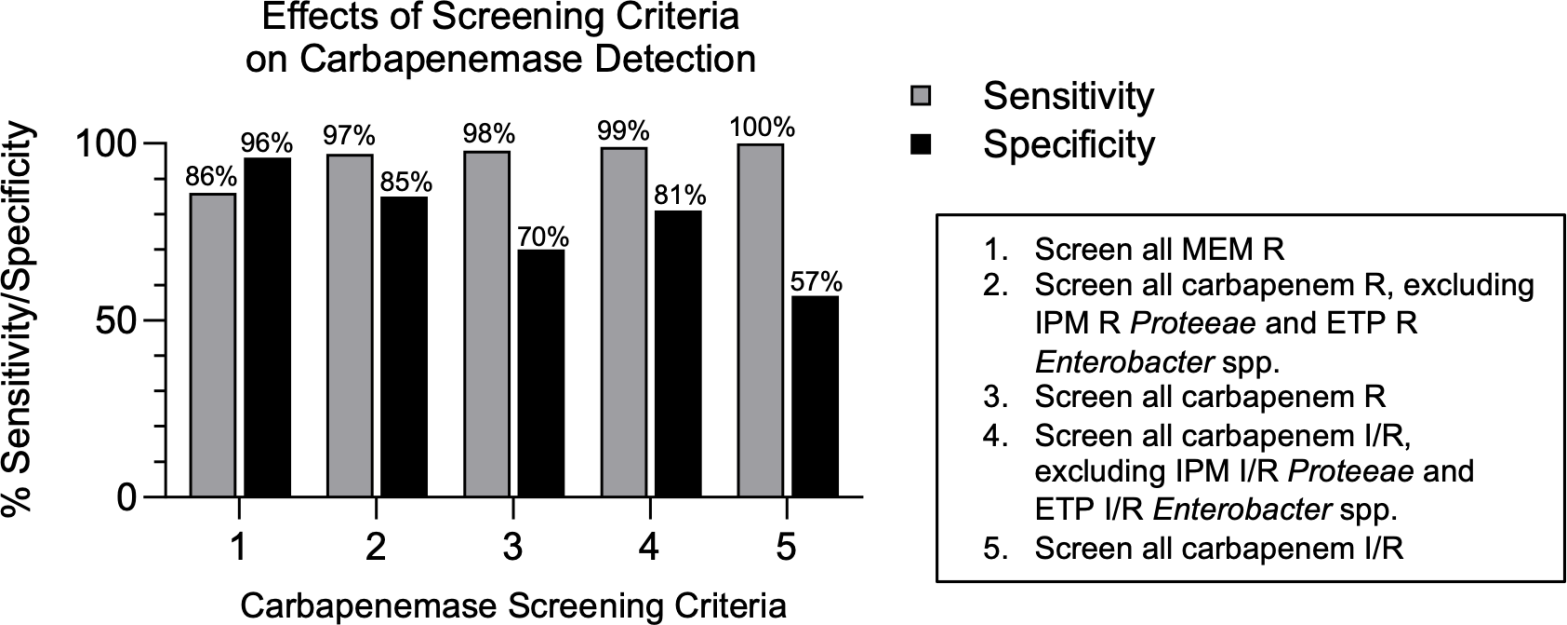
Effects of screening criteria on the sensitivity (gray) and specificity (black) of carbapenemase detection (*n* = 196). The number on the *x*-axis refers to the screening criteria described in the legend (right). ETP, ertapenem; IPM, imipenem; I, intermediate; MEM, meropenem; R, resistant; S, susceptible.

 VITEK2 susceptibility testing was performed on a challenge set of 200 clinical *Enterobacterales* with 196 analyzed after exclusion of 4 isolates due to discrepant or incomplete enrollment data. The study isolates represent a range of carbapenem resistance profiles, including 57 non-CRE, 24 non-CP-CRE, and 115 CP-CRE isolates (Table 1). As demonstrated by this set of isolates, resistance to a single carbapenem does not reliably identify all CP-CRE. For example, if only meropenem-resistant isolates were screened further for carbapenemase production, 14% (16 out of 115) of CP-CREs in this challenge set would be missed, including 4 KPC, 3 NDM, 8 OXA-48-like, and 1 IMP-27-producing isolates (Table 1, Fig. 1). Therefore, clinical laboratories must assess various carbapenemase screening criteria, considering the risks of undetected CP-CRE, testing costs, and workflow demands on the laboratory. The most conservative screening criteria considered involves screening all isolates that test intermediate or resistant to a carbapenem. This strategy maximizes sensitivity (100%) for detection of CP-CRE while increasing unnecessary screening of non-CP-CRE isolates (specificity = 57%) (Fig. 1). Strategies to increase the specificity of carbapenemase screening include excluding isolates less likely to be CP-CRE. Excluding *Proteeae* that test intermediate or resistant to imipenem and *Enterobacter* isolates that test intermediate or resistant to ertapenem increases screening specificity to 81%, though rare CP-CREs may be undetected. The effects of various screening criteria on the detection of CP-CRE for the isolate cohort are summarized in Fig. 1.

**TABLE 1:** Carbapenem resistance profiles of the study isolates by VITEK2 susceptibility testing^*a*^

Isolate group	No. of isolates	Imipenem			Ertapenem			Meropenem		
		S	I	R	S	I	R	S	I	R
Non-CRE	57	49 (86%)	8 (14%)	0 (0%)	53 (93%)	4 (7%)	0 (0%)	57 (100%)	0 (0%)	0 (0%)
WT	13	9 (69%)	4 (31%)	0 (0%)	13 (100%)	0 (0%)	0 (0%)	13 (100%)	0 (0%)	0 (0%)
ESBL/AmpC	44	40 (91%)	4 (9%)	0 (0%)	40 (91%)	4 (9%)	0 (0%)	44 (100%)	0 (0%)	0 (0%)
Non-CP-CRE	24	8 (33%)	3 (13%)	13 (54%)	6 (25%)	0 (0%)	18 (75%)	19 (79%)	2 (8%)	3 (13%)
CP-CRE	115	6 (5%)	4 (3%)	105 (91%)	4 (3%)	6 (5%)	105 (91%)	11 (10%)	5 (4%)	99 (86%)
KPC	58	1 (2%)	0 (0%)	57 (98%)	2 (3%)	5 (9%)	51 (88%)	3 (5%)	1 (2%)	54 (93%)
NDM	32	0 (0%)	0 (0%)	32 (100%)	0 (0%)	1 (3%)	31 (97%)	0 (0%)	3 (9%)	29 (91%)
OXA-48-like	10	5 (50%)	4 (40%)	1 (10%)	1 (10%)	0 (0%)	9 (90%)	7 (70%)	1 (10%)	2 (20%)
NDM, OXA-48-like	9	0 (0%)	0 (0%)	9 (100%)	0 (0%)	0 (0%)	9 (100%)	0 (0%)	0 (0%)	9 (100%)
SME	3	0 (0%)	0 (0%)	3 (100%)	0 (0%)	0 (0%)	3 (100%)	0 (0%)	0 (0%)	3 (100%)
IMI-1	1	0 (0%)	0 (0%)	1 (100%)	0 (0%)	0 (0%)	1 (100%)	0 (0%)	0 (0%)	1 (100%)
NDM and KPC	1	0 (0%)	0 (0%)	1 (100%)	0 (0%)	0 (0%)	1 (100%)	0 (0%)	0 (0%)	1 (100%)
IMP-27	1	0 (0%)	0 (0%)	1 (100%)	1 (100%)	0 (0%)	0 (0%)	1 (100%)	0 (0%)	0 (0%)
Total	196	63 (32%)	15 (8%)	118 (60%)	63 (32%)	10 (5%)	123 (63%)	87 (44%)	7 (4%)	102 (52%)

^*a*^ CP-CRE, carbapenemase-producing carbapenem-resistant *Enterobacterales*; CRE, carbapenem-resistant *Enterobacterales*; I intermediate; S, susceptible, R, resistant.

### Performance of the current VITEK2 AES for detection of CP-CRE

The bioART expert rules for predicting carbapenemase classes were designed to be used in conjunction with the VITEK2 AES. Before evaluating the performance of the bioART rules, the performance of the AES was analyzed for this cohort. AES characterization of resistance mechanisms for the study isolates is summarized in Table 2. Overall, the AES detected carbapenemase-producing isolates with a sensitivity of 83% (95 out of 115) and a specificity of 85% (69 out of 81). The 20 CP-CRE isolates not detected by the AES included 14 KPC^+^, 2 OXA-48-like^+^, 2 NDM^+^, 1 NDM^+^/KPC^+^, and 1 IMP-27^+^, which all had inconsistent AES reports. All 20 not detected CP-CREs tested as intermediate or resistant to at least one carbapenem, and 14 out of 20 isolates tested resistant to all three carbapenems tested by VITEK2 AST. There were 12 false-positive CP-CRE predictions by the AES, including 11 non-CP-CRE isolates and 1 ESBL/AmpC isolate. Overall, for this challenge set of isolates, the AES had a positive predictive value of 89% (95 out of 107) and a negative predictive value of 78% (69 out of 89) for CP-CRE detection.

**TABLE 2:** AES characterization of resistance mechanisms for the 196 study isolates

Isolate group	No. of isolates	AES characterization			
		WT	ESBL/AmpC-producing ± impermeability	Carbapenemase-producing ± impermeability	Inconsistent
Non-CRE	57	12 (21%)	37 (65%)	1 (2%)	7 (12%)
WT	13	12 (92%)	0 (0%)	0 (0%)	1 (8%)
ESBL/AmpC	44	0 (0%)	37 (84%)	1 (2%)	6 (14%)
Non-CP-CRE	24	0 (0%)	8 (33%)	11 (46%)	5 (21%)
CP-CRE	115	0 (0%)	0 (0%)	95 (83%)	20 (17%)
KPC	58	0 (0%)	0 (0%)	44 (76%)	14 (24%)
NDM	32	0 (0%)	0 (0%)	30 (94%)	2 (6%)
OXA-48-like	10	0 (0%)	0 (0%)	8 (80%)	2 (20%)
NDM, OXA-48-like	9	0 (0%)	0 (0%)	9 (100%)	0 (0%)
SME	3	0 (0%)	0 (0%)	3 (100%)	0 (0%)
IMI-1	1	0 (0%)	0 (0%)	1 (100%)	0 (0%)
NDM, KPC	1	0 (0%)	0 (0%)	0 (0%)	1 (100%)
IMP-27	1	0 (0%)	0 (0%)	0 (0%)	1 (100%)
Total	196	12 (6%)	45 (23%)	107 (55%)	32 (16%)

### Combined performance of the AES and prototype bioART rules at carbapenemase class identification

The performance of the AES combined with the prototype bioART rules for predicting MBL, KPC, OXA-48-like, and SME carbapenemase production was assessed for 158 of the 196 study isolates. Notably, SME carbapenemases are detected by the AES alone. Thirty-eight study isolates were excluded from this analysis because the bioART rules do not have claimed indications for use with the bacterial species (16 *Proteus* spp., 13 *Citrobacter* spp. other than *C. freundii* complex, 6 *Morganella* spp., 2 *Providencia* spp., and 1 *Kluyvera* spp.). For these 38 isolates, the AES detected carbapenemase production with a sensitivity of 76% (16 out of 21) and a specificity of 100% (17 out of 17). Further characterization of the carbapenemase class for these species cannot be achieved with the AES and bioART expert rules as currently designed.

For the remaining 158 study isolates, for which the bioART rules have claimed indications, the results were interpreted together with the AES predictions (Table 3). The cohort analyzed included 8 WT, 39 ESBL/AmpC, 17 non-CP-CRE, and 94 CP-CRE isolates. The CP-CRE isolates included 41 KPC+, 30 NDM+, 10 OXA-48-like+, 9 NDM/OXA-48-like+, 3 SME+, and 1 IMI-1+ isolates. Results for carbapenemase class detection by the AES and bioART rules are summarized (Table 4, top). The best performance was observed for prediction of SME and MBL carbapenemases, with 3 out of 3 (100%) SMEs and 38 out of 39 (97%) MBL carbapenemases detected. The one MBL that was not detected was an NDM-producing *Klebsiella pneumoniae* isolate for which no bioART prediction was made. The isolate tested resistant to all β-lactams by VITEK2 AST. The sensitivity for KPC carbapenemases was 76% (31 out of 41). Of the 10 KPC carbapenemases that were not detected, the bioART rules predicted 4 as OXA-48-like, 4 as MBL, and 2 with no bioART prediction. Sensitivity for OXA-48-like carbapenemases was 47% (9 out of 19 detected). However, of the 10 OXA-48-like-producing isolates that were not detected, 9 were dual carbapenemase producers that also encoded an NDM carbapenemase. All nine dual NDM^+^/OXA-48-like^+^ isolates were predicted to have an MBL carbapenemase. For isolates only encoding an OXA-48-like carbapenemase, sensitivity for OXA-48-like carbapenemases was 90% (9 out of 10) (Table 4, top). The not detected OXA-48-like^+^ isolate was predicted as either a KPC or MBL producer.

**TABLE 3:** Combined interpretations of the AES and bioART reports for characterization of resistance mechanisms^*a*^

AES report	bioART report	Combined AES + bioART interpretation	No. of isolates
WT	Any	WT	7
ESBL/AmpC ± impermeability	Any	ESBL/AmpC	37
Carbapenemase (SME) ± impermeability	Any	SME	3
Carbapenemase ± impermeability	Possibility of KPC carbapenemase	KPC	2
	Possibility of OXA-48-like carbapenemase	OXA-48-like	8
	Possibility of MBL carbapenemase	MBL	43
	Possibility of KPC carbapenemase, possibility of OXA-48-like carbapenemase	KPC or OXA-48-like	30
	Possibility of KPC carbapenemase, possibility of MBL carbapenemase	KPC or MBL	1
	Possibility of OXA-48-like carbapenemase, possibility of MBL carbapenemase	OXA-48-like or MBL	0
	None	Carbapenemase other than KPC, MBL, or OXA-48-like	3
Inconsistent	Possibility of KPC carbapenemase	KPC	1
	Possibility of OXA-48-like carbapenemase	OXA-48-like	3
	Possibility of MBL carbapenemase	MBL	1
	Possibility of KPC carbapenemase, possibility of OXA-48-like carbapenemase	KPC or OXA-48-like	12
	Possibility of KPC carbapenemase, possibility of MBL carbapenemase	KPC or MBL	0
	Possibility of OXA-48-like carbapenemase, possibility of MBL carbapenemase	OXA-48-like or MBL	0
	None	Inconsistent	7

^*a*^ The number of study isolates for which each scenario applied is listed in the right column (*N* = 158).

**TABLE 4:** Performance of carbapenemase class characterization by the Vitek 2 AES and bioART expert rules before (top) and after (bottom) incorporating an additional rule applying MEM/MEV MIC ratio to resolve isolates predicted as both KPC and OXA-48-like producers (*n* = 158)^*a*^

Carbapenemase class	No. of isolates	Sensitivity	Specificity	PPV	NPV
Without MEM/MEV MIC ratios to resolve isolates predicted to have both KPC and OXA-48-like enzymes					
MBL	39	97% (38/39)	93% (111/119)	83% (38/46)	99% (111/112)
KPC	41	76% (31/41)	87% (102/117)	67% (31/46)	91% (102/112)
OXA-48-like	19	47% (9/19)	68% (95/139)	17% (9/53)	90% (95/105)
OXA-48-like	10	90% (9/10)			
Dual NDM/OXA-48-like	9	0% (0/9)			
SME	3	100% (3/3)	100% (155/155)	100% (3/3)	100% (155/155)
Incorporating MEM/MEV MIC ratios to resolve isolates predicted to have both KPC and OXA-48-like enzymes					
MBL	39	97% (38/39)	93% (111/119)	83% (38/46)	99% (111/112)
KPC	41	71% (29/41)	95% (111/117)	83% (29/35)	90% (111/123)
OXA-48-like	19	47% (9/19)	91% (126/139)	41% (9/22)	93% (126/136)
OXA-48-like	10	90% (9/10)			
Dual NDM/OXA-48-like	9	0% (0/9)			
SME	3	100% (3/3)	100% (155/155)	100% (3/3)	100% (155/155)

^*a*^ NPV, negative predictive value; PPV, positive predictive value.

### Use of meropenem/meropenem-vaborbactam MIC ratios to differentiate KPC and OXA-48-like-producing isolates

An opportunity for improvement in carbapenemase class prediction by the AES and bioART rules was the specificity for KPC (87%) and OXA-48-like (68%) carbapenemases (Table 4, top). Forty-two isolates were predicted to produce both a KPC and an OXA-48-like carbapenemase, though no isolates enrolled in the study were dual KPC^+^/OXA-48-like^+^ isolates. A potential strategy to differentiate KPC and OXA-48-like producers is to assess whether meropenem-vaborbactam shows increased activity compared to meropenem alone. Vaborbactam is a β-lactamase inhibitor that is effective against class A KPC carbapenemases but does not inhibit class D OXA-48-like carbapenemases (19–21).

Of 29 KPC-producing isolates predicted to have both KPC and OXA-48-like carbapenemases, 93% (27 out of 29) had a MEM/MEV MIC ratio of ≥8. In contrast, 0% (0 out of 5) of the OXA-48-like isolates predicted to have both a KPC and OXA-48-like enzyme had a MEM/MEV MIC ratio ≥8. Of eight additional isolates predicted to have both KPC and OXA-48-like carbapenemases, three out of six non-CP-CRE, one out of one IMI-1, and zero out of one ESBL/AmpC had a MEM/MEV MIC ratio of ≥8. Based on these findings, MEM/MEV ratios were calculated to resolve isolates predicted as both KPC and OXA-48-like producers. A MEM/MEV MIC ratio of ≥8 was used to predict a KPC carbapenemase and exclude the possibility of an OXA-48-like enzyme. Conversely, a MEM/MEV MIC ratio of <8 predicted an OXA-48-like carbapenemase and excluded the possibility of a KPC carbapenemase. Clinical laboratories utilizing the AES and bioART rules for carbapenemase class detection could similarly calculate MEM/MEV ratios to differentiate KPC and OXA-48-like isolates. Table S3 depicts the modified interpretations of the AES and bioART results.

Overall performance of the AES and bioART rules for carbapenemase class detection after incorporation of MEM/MEV MIC ratios is shown (Table 4, bottom). The specificity of KPC detection improved to 95% (111 out of 117), and the specificity of OXA-48-like detection improved to 91% (126 out of 139). Because two KPC isolates had a MEM/MEV MIC ratio of <8, sensitivity for KPC carbapenemases decreased to 71% (29 out of 41).

### Analysis of the VITEK2 AES and bioART rules as tools in carbapenemase screening workflows

**Fig 2:**
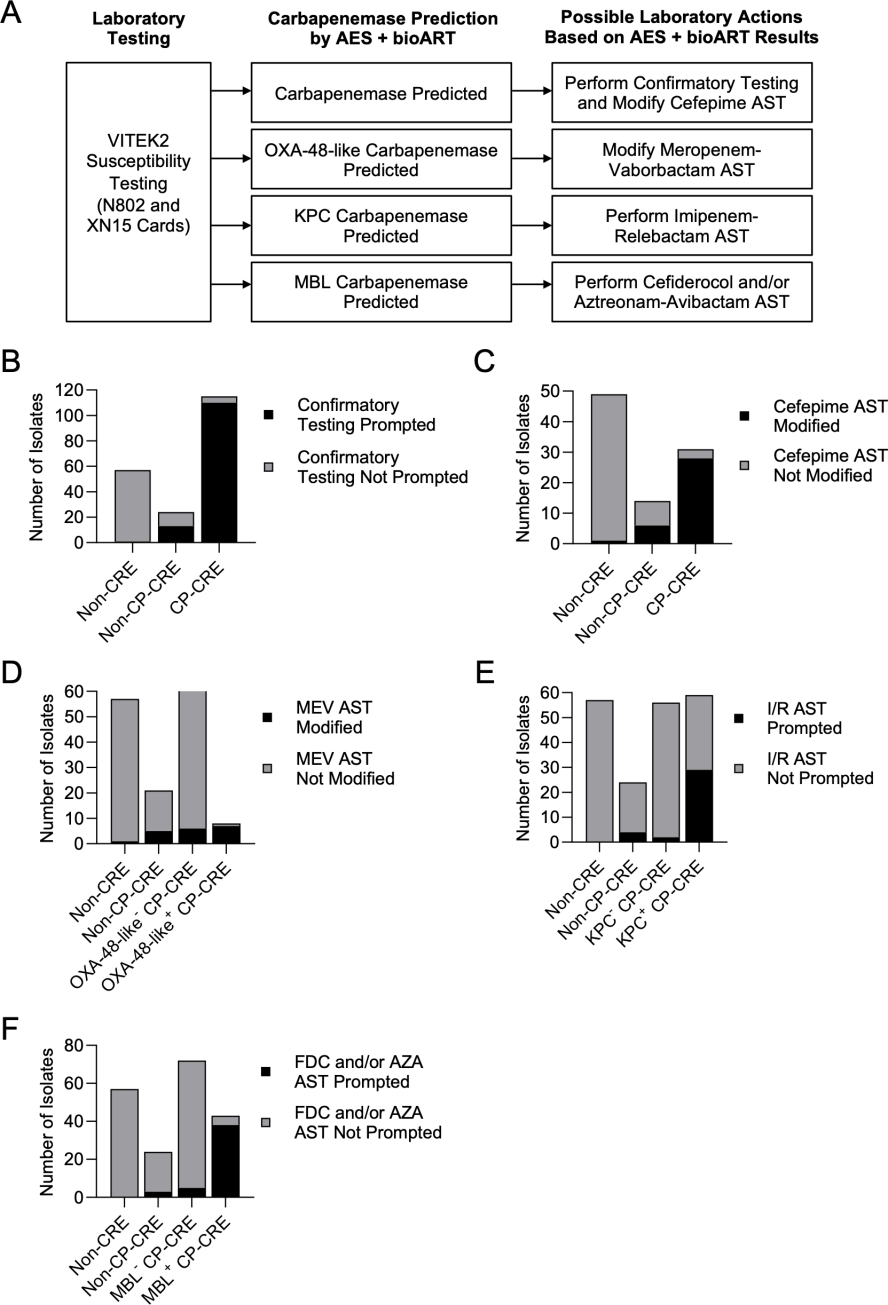
Analysis of the AES and bioART rules as tools in carbapenemase screening workflows for the study cohort (*n* = 196). (**B–F**) Outcomes of laboratory actions prompted by the AES and bioART rules, as described in panel **A**, for (**B**) prompting confirmatory carbapenemase testing, (**C**) modifying cefepime AST reporting, (**D**) modifying meropenem-vaborbactam AST reporting, (**E**) prompting reflex imipenem-relebactam AST, and (**F**) prompting reflex cefiderocol and/or aztreonam-avibactam AST. AZA, aztreonam-avibactam; I/R, imipenem-relebactam; FDC, cefiderocol; MEV, meropenem-vaborbactam.

The AES and bioART rules may serve as tools that can be integrated in carbapenemase screening workflows (Fig. 2A). For the complete study cohort of 196 isolates, when the AES and bioART predictions are used to prompt confirmatory carbapenemase testing, confirmatory testing would be performed for 2% of non-CRE (1 out of 57), 54% (13 out of 24) of non-CP-CRE, and 96% (110 out of 115) of CP-CRE (Fig. 2B). The five undetected CP-CREs included two KPC+, one NDM+, one NDM/KPC+, and one IMP-27+ isolate. Considering all 196 study isolates, the AES and bioART rules were more sensitive for carbapenemase detection than the AES alone. The AES alone would prompt confirmatory testing for 2% (1 out of 57) of non-CREs, 46% (11 out of 24) of non-CP-CREs, and 83% (95 out of 115) of CP-CREs. Overall, the sensitivity (96%) and specificity (83%) of the combined AES and bioART rules for CP-CRE detection are comparable to other phenotypic AST criteria clinical laboratories may use for carbapenemase screening (Fig. 1).

CLSI M100 guidelines recommend modifying or suppressing cefepime-susceptible or susceptible dose-dependent results for CP-CRE to discourage clinical use of the drug, especially for KPC-producing organisms (18, 22). Additionally, susceptible or intermediate meropenem-vaborbactam results are recommended to be suppressed or reported as resistant for OXA-48-like-producing isolates (18). If used to guide AST report modifications, the AES and bioART rules would prompt appropriate modifications of cefepime reports for 90% (28 out of 31) of CP-CREs that tested cefepime susceptible or susceptible dose dependent (Fig. 2C). In cases when a carbapenemase gene was incorrectly predicted, cefepime would be inappropriately modified for 2% (1 out of 49) of non-CREs and 43% (6 out of 14) of non-CP-CREs that tested cefepime susceptible or susceptible dose-dependent (Fig. 2C). Meropenem-vaborbactam tested as susceptible or intermediate for 80% (8 out of 10) of OXA-48-like isolates and for 0% (0 out of 9) of dual NDM/OXA-48-like-producing isolates. The AES and bioART rules would prompt modification of meropenem-vaborbactam results for 88% (seven out of eight) of meropenem-vaborbactam susceptible or intermediate OXA-48-like isolates (Fig. 2D). However, false-positive predictions of an OXA-48-like carbapenemase by the AES and bioART rules would lead to modification of meropenem-vaborbactam reporting for 2% (1 out of 57) of non-CREs, 24% (5 out of 21) of non-CP-CREs, and 10% (6 out of 62) of non-OXA-48-like CP-CRE isolates that tested meropenem-vaborbactam susceptible or intermediate, all of which were KPC+ isolates (Fig. 2D).

 Lastly, carbapenemase class prediction by the AES and bioART rules could serve as a tool to prompt additional AST. Antimicrobials used to treat CP-CRE infections that are not included on the N802 and XN15 panels include imipenem-relebactam that could be prompted for KPC isolates and cefiderocol and/or aztreonam-avibactam that could be prompted for MBL isolates (7). In this study cohort, the AES and bioART predictions would prompt imipenem-relebactam AST for 49% (29 out of 59) of KPC isolates. Imipenem-relebactam AST would also be prompted for 17% (4 out of 24) of non-CP-CREs and 4% (2 out of 56) of non-KPC CP-CREs, including one OXA-48-like^+^ isolate and one IMI-1^+^ isolate (Fig. 2E). Cefiderocol and/or aztreonam-avibactam testing would be prompted for 88% (38 out of 43) of MBL isolates in the study cohort. Testing would also be prompted for 13% (3/24) of non-CP-CREs and 7% (5 out of 72) of non-MBL CP-CREs, including four KPC^+^ isolates and one OXA-48-like^+^ isolate (Fig. 2F).

## DISCUSSION

Clinical laboratories are recommended to perform testing for carbapenemase detection; however, a wide range of phenotypic and genotypic methods are available, which can be incorporated into various workflows (4). The optimal screening criteria and workflow vary by laboratory and are influenced by regional carbapenemase epidemiology, patient population needs, and laboratory capabilities. When selecting carbapenemase detection methods, laboratories must consider test performance, complexity, and cost. This study assessed the performance of the VITEK2 AES and prototype bioART expert rules for detecting and characterizing CP-CRE and explored the integration of these tools into carbapenemase screening workflows. The prototype bioART rules analyzed were developed by bioMérieux and are currently available for laboratories that choose to implement them.

A key strength of this study was performance of WGS to identify less common carbapenemase genes. Additionally, a challenging isolate cohort that included 115 CP-CREs was tested to rigorously assess the capabilities of the AES and bioART rules. However, because a challenge set was used, the positive and negative predictive values reported here may differ from those observed in routine clinical practice, where carbapenemase prevalence varies by setting. A limitation of the study was the low number of OXA-48-like, SME-, IMP-, and IMI-producing isolates and absence of VIM-producing isolates, which limited the robust evaluation of these tools for detecting these enzyme classes.

For the study cohort, the combined AES and bioART rules resulted in detection of CP-CRE with a sensitivity of 96% and a specificity of 83%. The improved performance compared to the AES alone is attributed to the bioART rules’ independent ability to predict carbapenemase classes, even when the AES identifies an inconsistent phenotype. More challenging was the prediction of specific carbapenemase classes. MBLs were detected with a sensitivity of 97%, whereas sensitivity for detection of KPC enzymes was 76%. The sensitivity for OXA-48-like^+^ CP-CRE was 90%, whereas OXA-48-like carbapenemase production was undetected in OXA-48-like^+^/NDM^+^ isolates. This highlights a limitation of phenotypic testing, as the presence of an NDM carbapenemase masked the detection of OXA-48-like enzyme production. Specificity varied by carbapenemase class and was improved for KPC and OXA-48-like carbapenemases with the calculation and incorporation of MEM/MEV MIC ratios. In summary, while these tools provide valuable insights for carbapenemase detection, performance varied by carbapenemase class, underlining the need for laboratories to consider the downstream effects on patient care and infection prevention.

A significant advantage of the AES and bioART rules is that CP-CRE detection and carbapenemase class predictions can be made without incurring additional costs or hands-on testing. The VITEK2 AST system can deliver AST results within 8–12 h and is widely used by clinical laboratories around the world (23). The only testing requirement for carbapenemase characterization by the AES and bioART rules is to perform AST using routine VITEK2 cards. In this study, the N802 and XN15 cards were tested, as both include antibiotics required for rule predictions. Following customization, the bioART rules may be applied to other card configurations; however, bioMérieux recommends testing ceftazidime-avibactam and either meropenem-vaborbactam or imipenem-relebactam for optimal performance. Depending on card availability, the AST results for temocillin, piperacillin-tazobactam, ceftazidime, cefotaxime, ceftriaxone, cefepime, aztreonam, ceftazidime-avibactam, ceftolozane-tazobactam, ertapenem, imipenem, meropenem, imipenem-relebactam, and meropenem-vaborbactam can potentially be used by the bioART rules for carbapenemase detection. For species with claimed indications, the AES and bioART results are reported with the AST results. However, the utility of these tools for CP-CRE detection and carbapenemase class predictions is limited to laboratories that use multiple VITEK2 cards as part of their workflows. Laboratories may choose to run both cards in parallel or reflex to the XN15 Advanced MDRO Panel or a similar card if resistance is observed. Interpretation of the combined AES and bioART rule reports is a manual process, as they function separately. If implemented, procedures for manual interpretation of the AES and bioART results, along with the calculation and interpretation of MEM/MEV MIC ratios for optimal performance, are necessary (Table S3). The manual process for interpreting the AES and bioART results may pose challenges for routine implementation. Laboratories should also be aware that the bioART rules do not have claimed indications for all *Enterobacterales* species, including *Proteus*, *Providencia*, *Morganella*, and *Citrobacter* spp. other than *C. freundii* complex. Finally, the AES and bioART rules are only designed to detect KPC, MBL, OXA-48-like, and SME carbapenemases. Less common carbapenemase genes may go undetected or be misclassified.

In addition to the MIC value provided by phenotypic susceptibility testing, studies have shown that knowledge of the specific carbapenemase enzyme produced further influences antibiotic choice and treatment outcomes (7, 24, 25). Tiered carbapenemase testing workflows or lack of in-house testing may introduce delays, and integration of the rapid predictions by the AES and bioART could aid in providing early insights into resistance mechanisms. While the AES and bioART rules are not FDA-cleared for direct reporting of carbapenemase class predictions, they can serve as laboratory tools in carbapenemase screening workflows. These tools can prompt a variety of actions, including confirmatory carbapenemase testing, modifications to AST reports, and/or additional AST. These actions may be particularly beneficial in settings where confirmatory testing is not readily available while awaiting reference laboratory results. By guiding laboratory reporting and targeted reflex testing, these rapid predictions may, in turn, contribute to earlier therapy optimization and promote antimicrobial stewardship, especially for MBL isolates for which the bioART rules performed best. The data from this study can help laboratories predict the expected performance of these tools if implemented in these ways.

The application of expert rules to AST data has previously been shown to be valuable for clinical laboratories, particularly for inferring susceptibility results, determining when results for inappropriate antibiotics should be suppressed, and guiding when results should be edited based on inferred resistance mechanisms (26, 27). The AES and bioART rules, like any method, have advantages and limitations. Before implementation, their performance, limitations, and potential impacts must be thoroughly evaluated in relation to the needs of the laboratory. These tools can provide rapid resistance predictions but should be integrated thoughtfully, considering their effects on workflow, patient management, and infection prevention.

## SUPPLEMENTAL MATERIAL

Supplemental Material jcm.00769-25-s0001.pdf Tables S2 and S3; Figure S1.

Table S1 jcm.00769-25-s0002.xlsx Summary of isolates tested.

ASM does not own the copyrights to Supplemental Material that may be linked to, or accessed through, an article. The authors have granted ASM a non-exclusive, world-wide license to publish the Supplemental Material files. Please contact the corresponding author directly for reuse.
