# Supplementary material for: Evaluation of expert rules for carbapenemase class identification in Enterobacterales isolates using the VITEK2 susceptibility testing platform
Source: J Clin Microbiol. 2025 Sep 19;63(10):e00769-25. doi: 10.1128/jcm.00769-25 (PMC12506011; doi:10.1128/jcm.00769-25)
Supplement: Supplemental Material — Tables S2 and S3; Figure S1. [file jcm.00769-25-s0001.pdf]

Table S2.

AES Analysis Group	Vitek 2 AES Reports
WT	• Wild<br>• Wild (penicillinase)<br>• Wild (penicillinase), acquired penicillinase<br>• Wild (cephalosporinase), acquired penicillinase<br>• Acquired penicillinase
ESBL/AmpC ± Impermeability	• Acquired cephalosporinase (except ACC-1)<br>• Cephalosporinase (AmpC)<br>• ESBL<br>• ESBL, acquired cephalosporinase (except ACC-1)<br>• ESBL, ESBL + impermeability (cephamycins)<br>• ESBL, high level case (AmpC)<br>• ESBL (CTX-M like)<br>• ESBL (CTX-M like), high level case (AmpC)<br>• Extended spectrum beta-lactamase<br>• Extended spectrum beta-lactamase, high level case (AmpC)<br>• High level case (AmpC)<br>• High level case (AmpC), extended spectrum beta-lactamase<br>• HL cephalosporinase (AmpC)<br>• Impermeability carba (+ESBL or +HL AmpC)
Carbapenemase ± Impermeability	• Carbapenemase (+ or - ESBL)<br>• Carbapenemase (+ or - ESBL), impermeability carba (+ESBL or +HL AmpC)<br>• Carbapenemase (SME-like)<br>• Impermeability carba (+ESBL or +HL AmpC), carbapenemase (+ or - ESBL)
Inconsistent	• Inconsistent, observed results do not match any phenotype in the AES knowledge base

Table S2. Categorization of AES reports (right) into four analysis groups (left).

Figure S1.

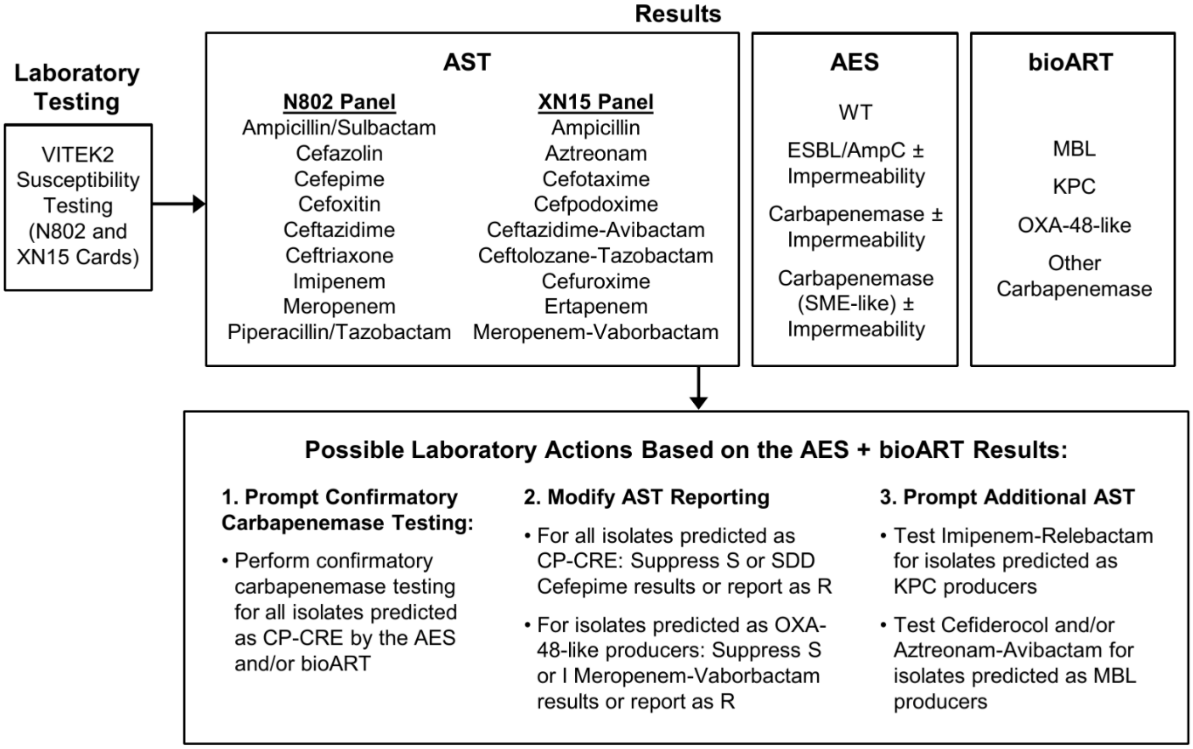

Figure S1. Laboratory workflow considerations for the VITEK2 AES and bioART tools.

Table S3.

AES Report	bioART Report	Meropenem/Meropenem-Vaborbactam MIC Ratio	Combined AES + bioART Interpretation
WT	Any	Any	WT
ESBL/AmpC ± Impermeability	Any	Any	ESBL/AmpC
Carbapenemase (SME) ± Impermeability	Any	Any	SME
Carbapenemase ± Impermeability	Possibility of KPC carbapenemase	Any	KPC
	Possibility of OXA-48-like carbapenemase	Any	OXA-48-like
	Possibility of MBL carbapenemase	Any	MBL
	Possibility of KPC carbapenemase, Possibility of OXA-48-like carbapenemase	≥8	KPC
		<8	OXA-48-like
	Possibility of KPC carbapenemase, Possibility of MBL carbapenemase	Any	KPC or MBL
	Possibility of OXA-48-like carbapenemase, Possibility of MBL carbapenemase	Any	OXA-48-like or MBL
	None	Any	Carbapenemase other than KPC, MBL, or OXA-48-like
Inconsistent	Possibility of KPC carbapenemase	Any	KPC
	Possibility of OXA-48-like carbapenemase	Any	OXA-48-like
	Possibility of MBL carbapenemase	Any	MBL
	Possibility of KPC carbapenemase, Possibility of OXA-48-like carbapenemase	≥8	KPC
		<8	OXA-48-like
	Possibility of KPC carbapenemase, Possibility of MBL carbapenemase	Any	KPC or MBL
	Possibility of OXA-48-like carbapenemase, Possibility of MBL carbapenemase	Any	OXA-48-like or MBL
	None	Any	Inconsistent

Table S3. Modified interpretations of the AES and bioART reports for characterization of resistance mechanisms. Meropenem/Meropenem-Vaborbactam MIC ratios can be calculated to differentiate isolates predicted as both KPC and OXA-48-like carbapenemase producers.
